# Chimpanzees (*Pan troglodytes*) Flexibly Adjust Their Behaviour in Order to Maximize Payoffs, Not to Conform to Majorities

**DOI:** 10.1371/journal.pone.0080945

**Published:** 2013-11-27

**Authors:** Edwin J. C. Van Leeuwen, Katherine A. Cronin, Sebastian Schütte, Josep Call, Daniel B. M. Haun

**Affiliations:** 1 Max Planck Research Group for Comparative Cognitive Anthropology, Max Planck Institute for Psycholinguistics, Nijmegen, The Netherlands; 2 Max Planck Institute for Evolutionary Anthropology, Leipzig, Germany; 3 Max Planck Research Group for Comparative Cognitive Anthropology, Max Planck Institute for Evolutionary Anthropology, Leipzig, Germany; 4 Max Planck Institute for Psycholinguistics, Nijmegen, The Netherlands; 5 University of Portsmouth, Portsmouth, United Kingdom; Centre national de la recherche scientifique, France

## Abstract

Chimpanzees have been shown to be adept learners, both individually and socially. Yet, sometimes their conservative nature seems to hamper the flexible adoption of superior alternatives, even to the extent that they persist in using entirely ineffective strategies. In this study, we investigated chimpanzees’ behavioural flexibility in two different conditions under which social animals have been predicted to abandon personal preferences and adopt alternative strategies: i) under influence of majority demonstrations (i.e. conformity), and ii) in the presence of superior reward contingencies (i.e. maximizing payoffs). Unlike previous nonhuman primate studies, this study disentangled the concept of conformity from the tendency to maintain one’s first-learned strategy. Studying captive (*n*=16) and semi-wild (*n*=12) chimpanzees in two complementary exchange paradigms, we found that chimpanzees did not abandon their behaviour in order to match the majority, but instead remained faithful to their first-learned strategy (Study 1a and 1b). However, the chimpanzees’ fidelity to their first-learned strategy was overridden by an experimental upgrade of the profitability of the alternative strategy (Study 2). We interpret our observations in terms of chimpanzees’ relative weighing of behavioural options as a function of situation-specific trade-offs. More specifically, contrary to previous findings, chimpanzees in our study abandoned their familiar behaviour to maximize payoffs, but not to conform to a majority.

## Introduction

 The capacity to flexibly switch between behavioural strategies might be the most critical means by which animals obtain and secure their competitive fitness advantage. Without the ability to abandon behaviour for better alternatives, animals would be dependent on the benevolence of external factors for whether they thrive or perish. Given the wide range of behavioural options available, animals are predicted to follow certain heuristics to optimize their behaviour [1]. One particular strategy that would increase an individual’s competitive advantage is the optimal foraging strategy, where individuals are expected to abandon their current behavioural patterns for more beneficial alternatives in order to maximize their net payoffs [2,3]. Similarly, animals may benefit from relying on the ‘wisdom of the crowd’, where they forgo personal strategies in order to match the strategy of the majority of group members [4]. This “conforming to majorities” can be beneficial because it allows subjects to quickly adopt locally adaptive strategies, especially in highly variable environments [5,6].

 Known for their inquisitive nature, chimpanzees (*Pan troglodytes*) display a rich palette of learning behaviour, both individually [7-9] and socially [10-12]. Moreover, chimpanzees display considerable between-group variation in behavioural patterns, many of which are understood in terms of social traditions (e.g. [13-15]). Yet, exactly how chimpanzees determine which behaviours to adopt and when to abandon their familiar practices for new ones (e.g. when environments change or when females migrate to other communities) is largely unknown. Studies focusing on potential majority influences have indicated that chimpanzees, like humans, may discount personal information in favour of the majority strategy [10,16,17]. In these studies, chimpanzees acquired one strategy socially, after which some individuals discovered the second, equally effortful strategy individually. The observation that the individual explorers reverted back to preferring the socially acquired information led researchers to conclude that chimpanzees showed “conformity” (see 18). However, this reversion paradigm has been criticized for leaving open alternative explanations, including persevering in using first-learned strategies [19,20], and for operationalizing conformity in terms of *maintaining* instead of *abandoning* familiar behaviour. This leaves open the question whether chimpanzees would flexibly *switch* strategies under the influence of majority demonstrations (see 20,21).

 Interestingly, chimpanzees have been shown to be rather conservative in different experimental designs where switching was rewarding. When chimpanzees were faced with a new challenge, their previous knowledge either hindered the acquisition of the more optimal solution [22,23], or prevented them from trying the novel (more rewarding) alternative [24,25]. This relative inflexibility seemed to persist even when their familiar behaviour was made entirely ineffective [24]. One criticism of these studies has been that the two strategies were not always structurally identical and thus might not have been equally effortful for the chimpanzees (see 16). In conjunction, these findings beg the question of under what circumstances chimpanzees would flexibly adjust their behaviour. This question sparked our goal of evaluating chimpanzees’ relative tendency to change behaviour under conditions of i) majority influences, and ii) superior reward contingencies. 

 Taken together, in this study, we investigated the extent to which chimpanzees are inclined to flexibly adjust their behaviour under two different conditions. First, we aimed to test whether minority chimpanzees would abandon their first-learned strategy for the conflicting majority strategy (Study 1). For this reason, we opted to operationalize the phenomenon of “conformity” as the tendency to *forgo previous knowledge* under influence of a majority of group members demonstrating an alternative strategy (human social psychology; e.g. [26]) rather than adopting the cultural evolutionary framework where *naïve individuals* are scrutinized for their tendency to copy the majority of group members with a disproportionate likelihood (e.g. [5]). Moreover, for validation purposes, we applied this operationalization (see *Methods*) in two different designs in two different chimpanzees populations (Study 1a and 1b). Second, we aimed to test whether chimpanzees would abandon their first-learned strategy when an equally effortful, yet superior reward contingency was present (Study 2). This study improved on earlier designs by testing the chimpanzees in their natural social group and having two structurally identical strategies available (avoiding strategy preferences based on relative ease of execution), where the only differences between the strategies were the location and profitability (cf. [24,25]). Moreover, extending prior research, we tested *learned* preferences rather than pre-established food preferences (cf. [16]).

## Methods

### Ethics Statement

 Research was performed in accordance with the recommendations of the Weatherall report ‘‘The use of nonhuman primates in research’’ [27]. All chimpanzees were fed a varied diet of fruits, vegetables and cereals and had ad libitum access to water. The normal diet was not restricted in this study and the chimpanzees gained extra food by participating. We certify that we have followed the rules as outlined in the “PASA Primate Veterinary Healthcare Manual,” that the research adhered to the ASAB/ABS Guidelines for the Use of Animals in Research, that all animal husbandry procedures were non-invasive and that participation by the animals was voluntary. The research protocols were approved by the Max Planck Institute for Evolutionary Anthropology Ethics Committee and the Chimfunshi Research Advisory Board.

### Study 1

 Study 1 consisted of two complementary designs. In both Study 1a & 1b the chimpanzees could exchange a token for a food reward. However, Study 1a used two token types exchanged at a single location to distinguish between the majority and minority strategy, whereas Study 1b used two spatially distinct locations and one token type. By using two different experimental designs and two chimpanzee populations, we aimed to increase the validity of our study and test whether minority chimpanzees forgo their first-learned behaviour for the strategy performed by the majority of group members.

### Study 1a: Wolfgang Kohler Primate Research Center

#### Housing and Study Subjects

This study was conducted at the Wolfgang Kohler Primate Research Center, Germany. The chimpanzees under study have access to an indoor (430 m^2^) and outdoor enclosure (4,000 m^2^). All enclosures include climbing structures, natural vegetation, and forms of enrichment (puzzle-boxes, jute bags, provisioning of concealed food). The group spends the nights in a series of sleeping rooms (47 m^2^). Subjects were 16 chimpanzees (5 males), ranging in age from 6-36 years. The trained majority consisted of 11 subjects (3 males; *M*_age_ = 22.8 years; range = 6-36 years), the minority comprised 5 subjects (2 males; *M*_age_ = 17.4 years; range = 7-35 years). The subgroups (majority and minority) were counterbalanced based on rank, age and sex as evenly as possible, except for one mother-offspring pair: mother (Ulla) and juvenile son (Kofi) were placed in the same subgroup based on recommendations from the chimpanzee keepers (both chimpanzees were allocated to the majority; see Table S1).

#### Procedure

**Figure 1:**
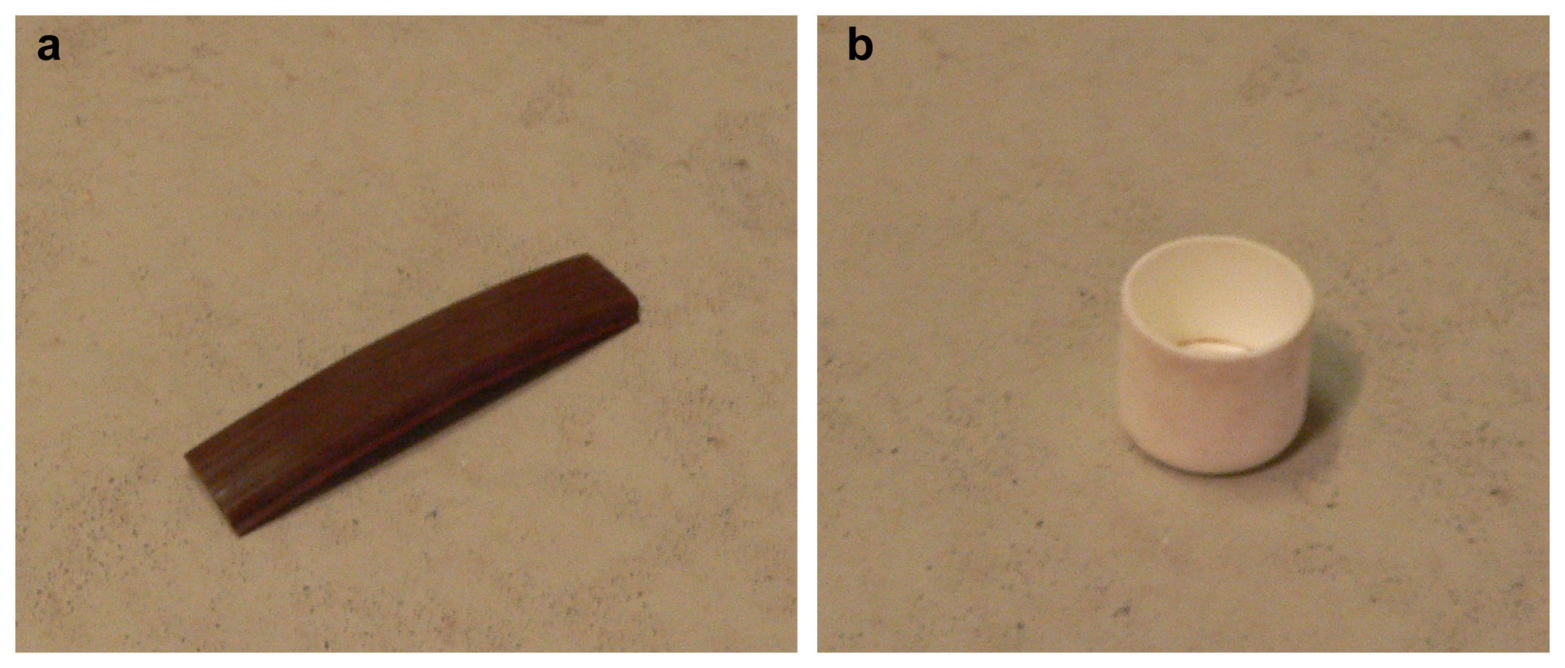
Depicted are the tokens that the subgroups in Study 1a were trained on: brown, plastic sticks for the majority (a), and white, plastic cups for the minority (b).

First, subjects were individually trained on a token-reward contingency, where the majority subjects were trained on brown, plastic sticks (Figure 1a) and the minority subjects on white, plastic cups (Figure 1b). Chimpanzees were presented with a token and rewarded one piece of apple for returning it to the experimenter. After the first exchange, 6 tokens were presented on a tray in an adjacent, accessible room. The chimpanzees had to collect the tokens, travel back to the experimenter and put them through a hole in a piece of perspex that was attached to safety mesh separating the chimpanzees from the experimenter. One training session consisted of exchanging a set of 6 tokens 3 consecutive times and lasted an average of 12.4 minutes (range 6.7 - 19.3 minutes). All chimpanzees were trained on their respective token for one session on three days over the course of two weeks, and returned all tokens successfully.

 Second, individuals were trained in their indoor enclosure with only their respective subgroup (majority/minority) present. For the first four sessions (one hour each, one per day), the human experimenter sat behind the safety mesh (the ‘exchange station’) and a token dispenser (containing only the trained token) was attached on the same mesh. The dispenser was made out of perspex and was continuously, automatically refilled. Chimpanzees could freely obtain tokens from the dispenser. For four subsequent sessions (one hour each, one per day), the dispenser was moved to a location approximately 30 meters from the exchange station. Throughout these eight training sessions, eight majority and two minority individuals participated, with each individual exchanging at least 20 tokens per session. Participation may have been less in these training sessions due to the increase in physical and social distractions compared to individual training.

**Figure 2:**
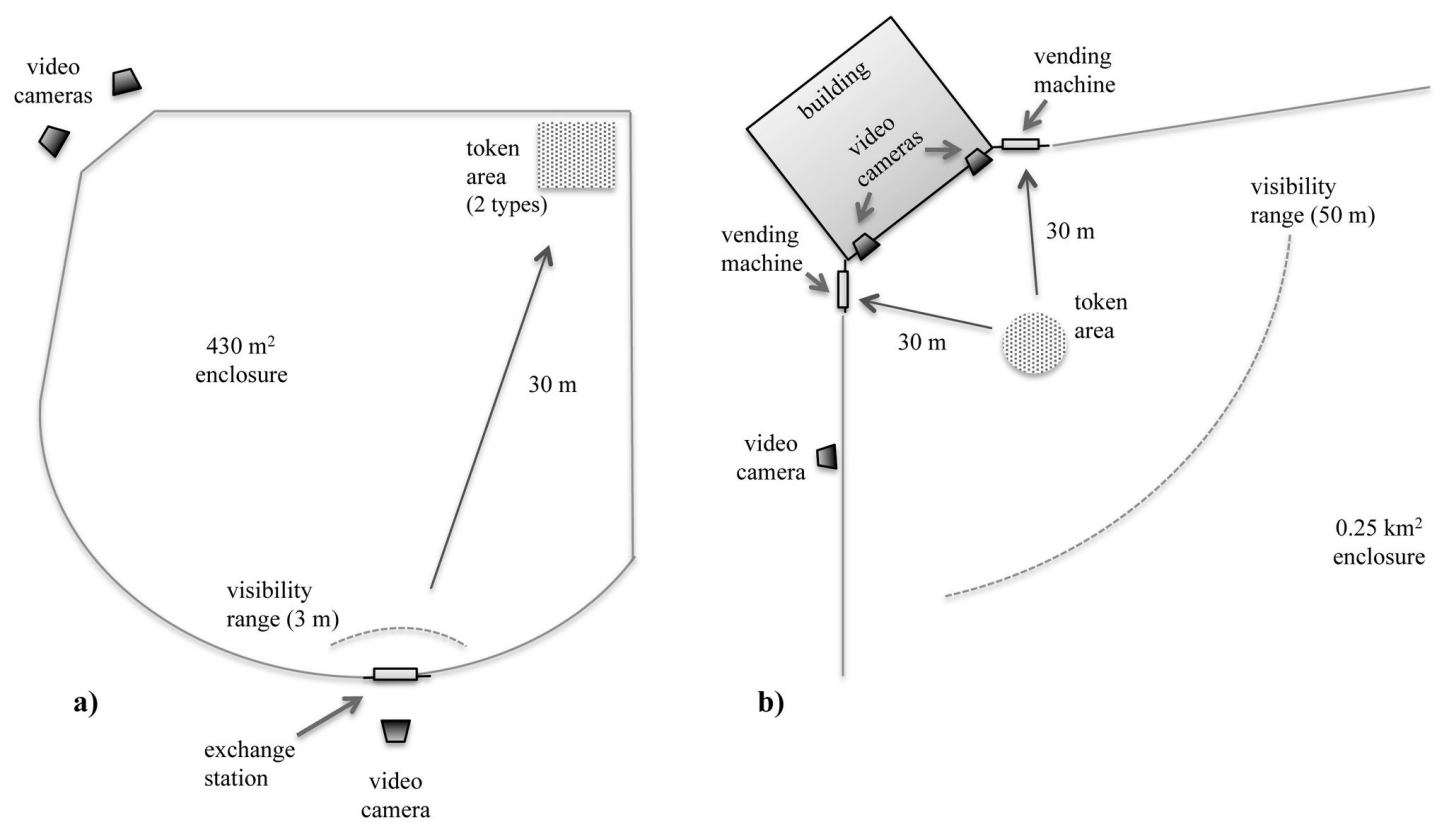
Schematic overview of the experimental setup in Study 1a, Leipzig Zoo (a) and Study 1b and 2, Chimfunshi Wildlife Orphanage Trust (b).

 During testing, both tokens (sticks and cups) were available at one location in the enclosure (from two dispensers, each containing one type of token) and rewarded equally (one piece of apple per token) upon delivery at the exchange station (see Figure 2a). Chimpanzees were tested for one hour on ten consecutive days in March 2012 (one week after training). All sessions were recorded using JVC GY-HM100U HD video cameras from three vantage points. Auditory commentary was provided on one of the JVC cameras by the experimenter (detailing which chimpanzee exchanged which token type). Auditory comments were subsequently used to extract information on the “token exchanges”, the videos were analysed for obtaining the “perception records”, i.e. for each individual within a 3-meter radius of the exchange station, we identified which other individuals were exchanging tokens and which type of token they used. This information amounted to individual scores of the number of times the majority and minority token had been observed to be used in exchanges, and the number of different subjects that were observed to use the two different token types. Whereas all tokens were equally rewarded upon exchanging, only the token exchanges where the individual had collected the token at the dispenser (*n*=2102) were included in the first analysis investigating the effect of majority demonstrations on the behaviour of minority chimpanzees; tokens that had been stolen from others (*n*=103) were considered opportunistically collected instead of chosen. A second analysis including all tokens (collected from dispenser and stolen, *n*=2205) was performed to investigate whether the pattern of results would differ.

### Study 1b: Chimfunshi Wildlife Orphanage Trust

#### Housing and Study Subjects

This study was conducted at the Chimfunshi Wildlife Orphanage Trust, a sanctuary that houses more than a hundred chimpanzees under close to natural conditions in the north-western part of Zambia (for details, see 14). The chimpanzees under study (Group 4) live in a 0.25 km^2^ enclosure of Miombo forest [28]. The chimpanzees spend all their time outside (including the nights), except for one 2-hour food-provisioning session per day, during which they receive additional fruits and vegetables in their indoor holding facility. Subjects were 12 chimpanzees (6 males), ranging in age from 4-21 years. The majority comprised 8 chimpanzees (3 males; *M*_age_ = 13.0 years; range = 4-18 years), the minority 4 chimpanzees (3 males; *M*_age_ = 14.8 years; range = 7-21 years). The subgroups (majority and minority) were counterbalanced based on rank, age and sex as evenly as possible (see Table S1).

#### Procedure

First, chimpanzees were individually trained on a token-reward contingency, where chimpanzees received one peanut for putting a wooden ball (Ø=3.0cm) through a hole in a piece of perspex that was attached to the mesh of their indoor holding space. At this stage, the balls were first handed to the chimpanzees and imitations of chimpanzee vocalizations were used to engage the chimpanzees. After the chimpanzees engaged readily, we threw the balls into their holding space and tested whether they would participate. Ten individuals (7 majority, 3 minority) reached the criterion of returning 10 balls through the hole in the perspex on at least three days; the remaining two individuals did not participate and were not included in the study.

 Second, subjects were trained in their outdoor enclosure with only their respective subgroup (majority/minority) and one vending machine present. During this subgroup training, the other subgroup remained inside the building without a clear view on the side where the other subgroup was being trained as to prevent any social learning from happening prior to testing. The vending machines comprised perspex construction (75x45x32 cm) supporting an automated food-dispenser with a metal front (painted as two Zambian bakeries: “Princes Bakery” for the majority, and “G&G Bakery” for the minority; see Figure S1). The machines were attached to safety mesh such that the chimpanzees could view the uniquely painted metal front; each front had an upper hole for token insertion and a lower hole to dispense food. Initially, an experimenter provided one piece of food through the lower hole of the machine after the chimpanzee had inserted a ball through the upper hole, later an automated device dispensed the food and the experimenter remained > 10 m away. Nine individuals (6 majority) reached the criterion of exchanging 10 balls on at least three days.

 We tested the entire social group in their outdoor enclosure for one hour on ten consecutive days in April 2012 (one week after training), during which both vending machines were available at approximately 20 meters from each other (see Figure 2b). Importantly, and contrary to the token design of Study 1a, we used this “spatially-distinct strategies design” in order to make it easier for the subjects to observe which strategies the other subjects were using. For the first six days, both vending machines were operational, yielding one peanut per ball automatically. Due to machine malfunctioning, during the final four testing days, experimenters manually controlled food dispensation through the machines (without being visible to the chimpanzees). The experimenters rewarded upon the audible click of a ball entering the vending machine and were not able to monitor the behaviour of the chimpanzees. Balls were thrown to the subjects in a randomized order (using a random name selection procedure without replacement) at a predefined distance of approximately 30 meters from each vending machine, with 1 or 2 balls per throw. Deviation from the randomized order sometimes occurred (e.g. when subjects were not present). Once one chimpanzee obtained one or more balls, the experimenter would wait for this individual to exchange the ball(s) and leave the vending machine before targeting another individual. This procedure was adopted to increase the likelihood that chimpanzees could choose between the vending machines without one of them being occupied by another individual. All sessions were recorded using JVC GY-HM100U HD video cameras from three vantage points. Auditory commentary was provided on one of the JVC cameras by the experimenter, detailing which chimpanzee exchanged at which vending machine and whether either machine was occupied by another individual. Auditory comments and videos were subsequently used to extract information on the “token exchanges”, videos were analysed for obtaining the “perception records”, i.e. the focal individual’s presence within visibility range (≤ 50 meters from the vending machines; see Figure 2b), while other subjects were making choices. Whereas all balls were equally rewarded upon insertion in the vending machines, only the exchanges that occurred when neither vending machine was occupied by another individual (*n*=413) were used for the analysis of whether the minority chimpanzees adjusted their behaviour to the majority for the reason that we were interested in the chimpanzees’ free strategy choices (not biased by the social inaccessibility of one of the vending machines). However, similar to Study 1a, in a subsequent analysis, we additionally used the full dataset (*n*=861) in our analysis of the behaviour of the minority chimpanzees. 

 For establishing the perception records, since exchanges could be visible regardless of whether the tokens were collected or stolen (Study 1a) or whether one or two vending machines were occupied (Study 1b), all exchanges were included (Study 1a: *n*=2205; Study 1b: *n*=861). All analyses were two-tailed unless indicated differently.

#### Results

All chimpanzees, both in the majority and minority, preferred to use their trained strategy over the course of 10 test days, both in Study 1a and 1b (one-sample Wilcoxon signed rank test against 50% (no preference for either strategy), Study 1a: *W*=2.82, *n*=10, *p*=0.005, median = 86.8%, range = 31.1 - 100%; Study 1b: *W*=2.71, *n*=9, *p*=0.007, median = 100%, range = 77.8 - 100%).

**Figure 3:**
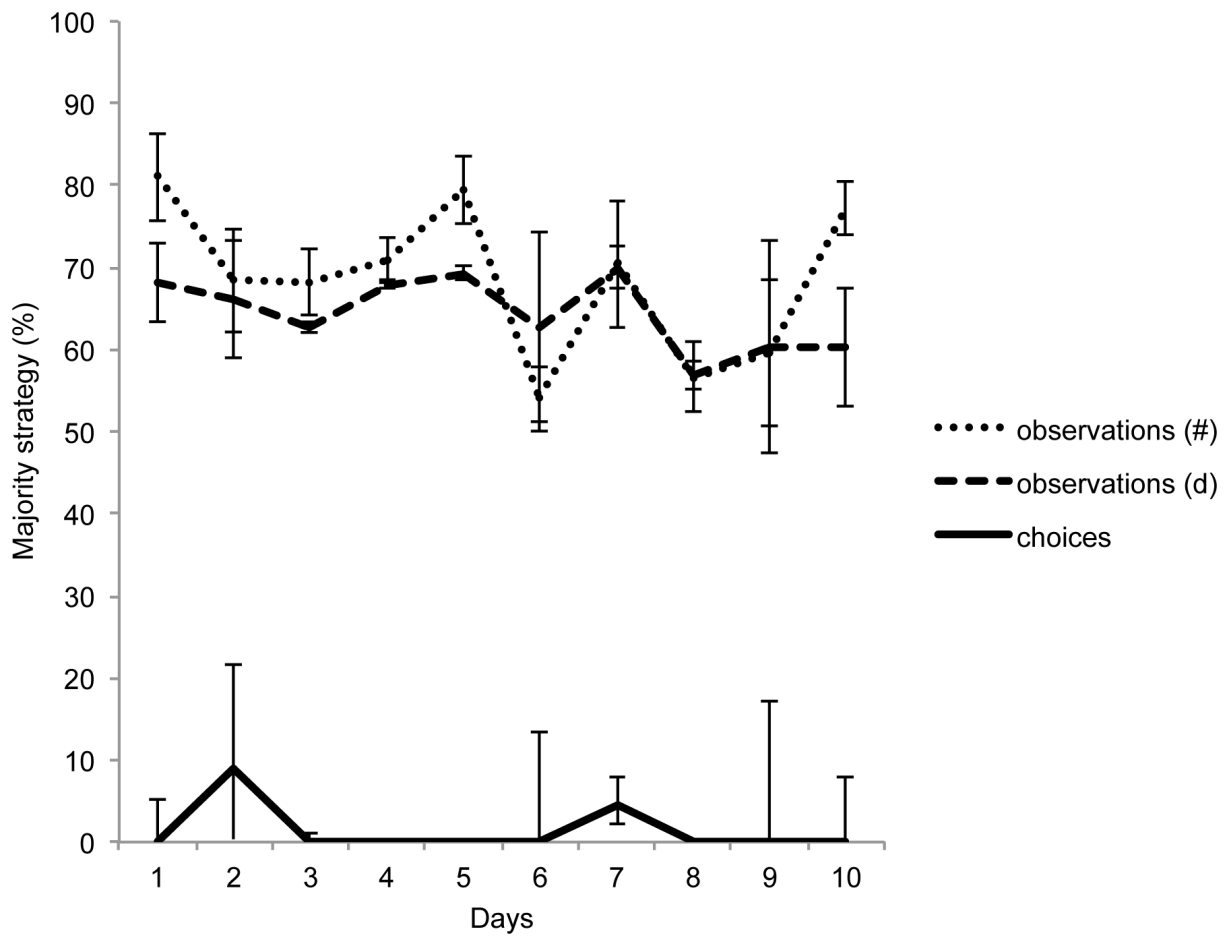
Mean (± s.e.m.) percentage by which the minority chimpanzees of Study 1 (n=5) *observed* majority strategy demonstrations, both in absolute frequencies (#) and in number of unique demonstrators (d), supplemented with the median percentages (with the lower and upper error hinge representing the first and third quartile, respectively) by which the minority chimpanzees *chose* to use the majority strategy per day (Exp. 1a: mean choices per individual per day = 39.9 token exchanges, range 38-103; Exp. 1b: mean choices per individual per day = 11.4 exchanges at the vending machines, range 7-23).

 Focusing in on the chimpanzees in the minorities, we found no evidence for conformity in either chimpanzee population. While the perception records indicated that minority chimpanzees more often observed chimpanzees using the majority strategy compared to the minority strategy, both in absolute frequency (regardless of which individual was exchanging) and in the number of unique individuals (see Figure 3), they remained faithful to their trained strategy with high fidelity (*W*=2.06, *n*=5, *p*=0.039; Figure 3), both in Study 1a (*n*=2, median = 99.1%, range = 67.9 - 100%, token exchanges per individual per day *M* = 38.7) and Study 1b (*n*=3, median = 100%, range = 33.3 - 100%, free exchanges at the vending machines per individual per day *M* = 8.7). See Table S2 for an overview of all token and vending machine exchanges of the minority chimpanzees in Study 1a and 1b.

 Analysis of all choices made by the minority chimpanzees (thus, including stolen tokens in Study 1a and location choices where one vending machine was already occupied by another chimpanzee in Study 1b) yielded similar results (fidelity to trained strategy: *W*=2.03, *n*=5, *p*=0.042), both in Study 1a (median = 98.8%, range = 68.8 - 100%) and 1b (median = 100%, range = 0 - 100%). 

### Study 2

#### Study Subjects & Procedure

Here, we investigated chimpanzees’ strategy use within an unequal reward paradigm. While employing the same procedure as in Study 1b – with the same individuals at the Chimfunshi Wildlife Orphanage Trust (Zambia) – the chimpanzees could now choose between a machine that yielded the same reward as in Study 1b (1 peanut/ball) or the alternative machine that yielded 5 peanuts/ball. Because this study was designed to test whether chimpanzees would change their behaviour upon the introduction of a superior alternative strategy, we maximized the number of individuals in our sample by upgrading the vending machine that was previously used by the least number of individuals (“G&G Bakery”). In other words, we aimed to investigate the behaviour of the chimpanzees that had been in the majority in Study 1b (n=6), leaving the minority chimpanzees of Study 1b (n=3) out of this sample. The chimpanzees were tested for 1 hour per day on 10 consecutive days (immediately following the end of Study 1b). Again, all exchanges were rewarded based on the predefined reward-paradigm (1 peanut/ball at “Princes Bakery” and 5 peanuts/ball at “G&G Bakery”). All sessions were recorded using JVC GY-HM100U HD video cameras from three vantage points. Videos were subsequently analysed for “vending machine choices”, where the choices of the chimpanzees who had a pre-existing preference for the machine that continued to provide a single reward were central to the analysis. In a first analysis, for the same reason as in Study 1b, only the exchanges where no machine was occupied (*n*=321) were used and a subsequent analysis included all exchanges (*n*=416). All analyses were two-tailed unless indicated differently.

#### Results

**Figure 4:**
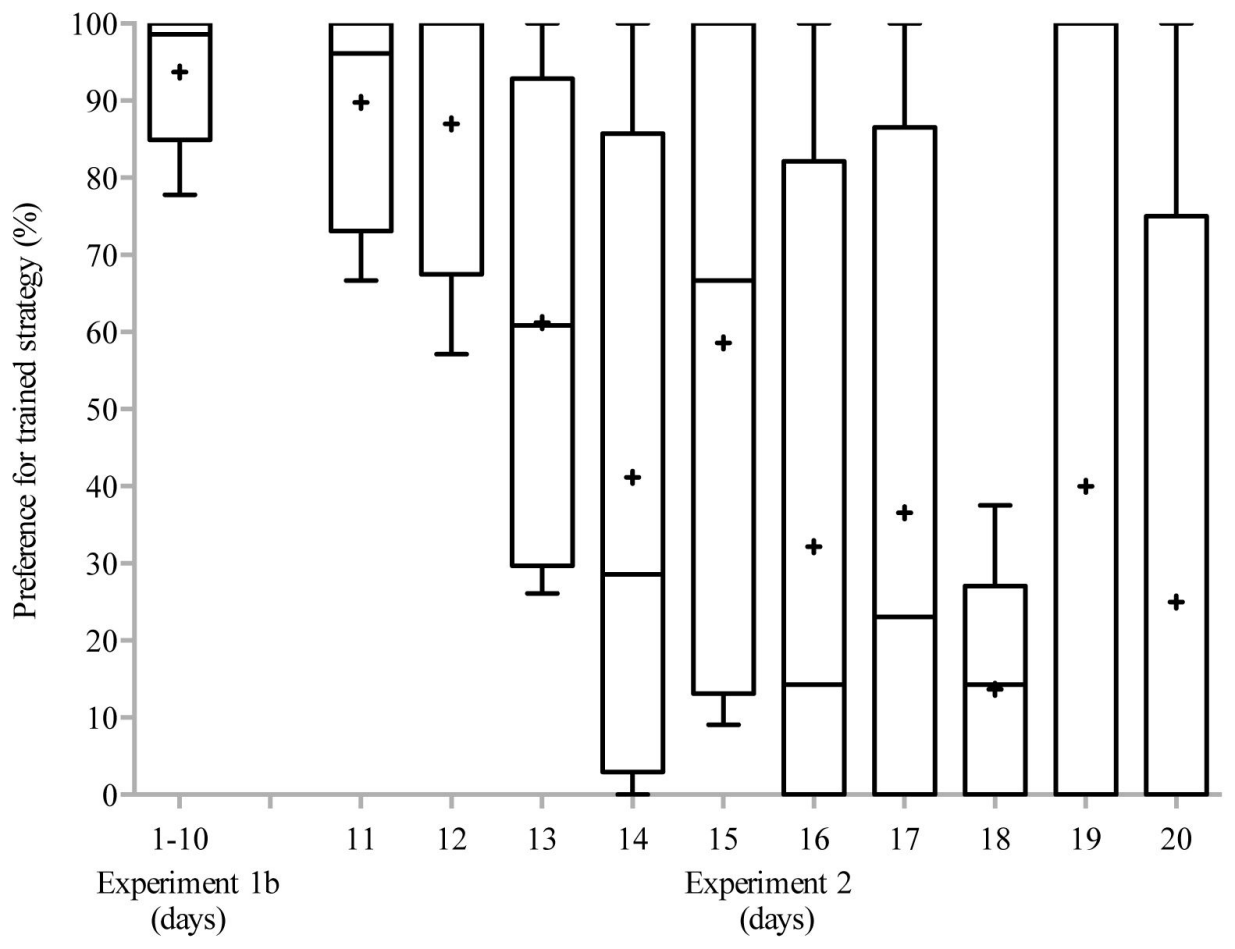
Median (with the lower and upper boxplot hinge representing the first and third quartile, respectively, and the crosses within the boxplots representing the means) preference for the trained strategy of the majority chimpanzees in Zambia throughout Study 2. Data point at time point “1-10” represent the median preference of the majority chimpanzees for the trained strategy over the first 10 days (Study 1b). Data points at time points 11 to 20 refer to the median preferences for the trained strategy (i.e. the least productive) in the ‘superior reward contingency’ design (Study 2).

Upon upgrading the alternative strategy, the majority chimpanzees of Study 1b started switching their strategy (see Figure 4), leading to a significant change of their preferences when comparing the 10 testing days of Study 1b to the subsequent 10 testing days in Study 2 (related-samples Wilcoxon signed rank test: *Z*= -2.02, *n*=6, *p*=0.043, median_1b_ = 100%, median_2_ = 37.8%). The same results were obtained when analysing all exchanges, including the ones where one or both of the vending machines were already occupied by another chimpanzees (*Z*= -2.02, *n*=6, *p*=0.043, median_1b_ = 100%, median_2_ = 41.6%). On the individual level, comparing the last session of Study 1b (henceforth “T1”) to the last session of Study 2 (henceforth “T2”), three chimpanzees had significantly changed their preference from their familiar strategy to the upgraded strategy (one-tailed Fisher exact tests with in subscript the choices for the trained and not-trained strategy, respectively: *Individual 1*: T1_6,0_ T2_3,15_ *p*<0.001; *Individual 2*: T1_17,0_ T2_0,12_ *p*<0.001; *Individual 4*: T1_9,0_ T2_1,6_ *p*<0.001; Bonferroni-Holm corrected *p*-value=0.008). Regarding the remaining chimpanzees: *Individual 3* started using the upgraded strategy (see Figure S2), where her behavioural choice on the last day that she engaged in the study indicates that she switched from her trained strategy to the upgraded strategy (day4_0,1_). However, due to absence of participation after day 4, no standardized analysis could be done. *Individual 5* did not switch strategies (T1_5,0_ T2_4,0_ *p*=1.0). However, this 7-year old male started switching strategies until day 8 (comparing T1_5,0_ to day8_2,5_ : *p*=0.027; Bonferroni-Holm corrected *p*-value=0.025), after which he reverted back to his trained strategy (see Figure S2). *Individual 6* never used the upgraded strategy (T1_8,0_ T2_6,0_ *p*=1.0; see Figure S2). Importantly, there was no indication that the strength by which these six chimpanzees had experienced their first-learned strategy throughout the preceding conformity study (Study 1b) related negatively to their switching behaviour in this upgraded paradigm: For instance, the two individuals that switched relatively quickly (Ind. 2 and 4; see Figure S2) had the most personal experience with their first-learned strategy in the conformity study (84 and 105 exchanges respectively; average across individuals = 46 exchanges), while they observed the usage of their first-learned strategy roughly as much as the other chimpanzees under scrutiny (312 and 336 exchanges respectively; average across individuals = 316 exchanges). The female that never switched in the upgraded paradigm (Ind. 6; see Figure S2) had an average experience with her first-learned strategy throughout the conformity study, both personally (53 exchanges) and socially (328 exchanges). 

## Discussion

 In this study, we investigated two possible conditions under which chimpanzees might flexibly adjust their familiar behaviour by sequentially exposing them to conflicting majority influences and superior payoff alternatives. When tested in the presence of a majority of individuals using an alternative, equally beneficial strategy, chimpanzees remained faithful to their first-learned strategy with high fidelity. However, when the chimpanzees’ strategy was made relatively inefficient by upgrading the yield of the alternative strategy, chimpanzees tended to forgo their first-learned strategy in favour of the more productive strategy. In conjunction, these results indicate that chimpanzees adjust their behaviour conditionally. In this study, where chimpanzees did not change their behaviour in order to conform to the majority of group members, the inclination to maximize personal benefits drove chimpanzees to adjust their behaviour. These findings stand in contrast to some recent social learning and conformity studies ([16,24,25]; but see 29,30).

 Notably, this study may provide an alternative explanation for the observation that chimpanzees tended to revert back to their first-learned strategies after discovering an equally or even more rewarding alternative strategy in previous studies [10,16,17]. In these studies, the behavioural pattern of reverting back to using the first-learned strategy was interpreted in terms of conformity (also see 18), even for reasons of ‘aiding social cohesion and the maintenance of group dynamics’ ([16], pp. 6). Hopper and colleagues [16] proposed to interpret the behaviour of the chimpanzees in their study as ‘normative conformity’, a term coined by Deutsch and Gerard [31] to distinguish conformity based on the desire to create or maintain a positive group sense (normative conformity) from conformity based on the aim to obtain the most fitting strategy in a given environment (informational conformity) [31]. However, in their study ([16]; but also see 10,17), the conformity strategy coincided with the strategy to persevere in first-learned practices, which makes the conclusion that the chimpanzees were conformists in the first place premature. The present study shows that chimpanzees remain faithful to their first-learned strategy, even when it is not the strategy used by most group members, which is indicative of a conservative tendency rather than conformity [20]. The fact that chimpanzees have been shown to be sensitive to majority demonstrations when acquiring *novel* behaviour [32] indicates that although the majority may represent a vector in the decision-making process of chimpanzees, it does not necessarily provide a strong enough incentive to make them change their behaviour. Moreover, in this study by Haun and colleagues focusing on majority influences in the context of acquiring novel behaviour (see 32), the most likely explanation in motivational terms would be that the chimpanzees use the demonstrations to obtain knowledge about their environment (informational conformity), as they were not exposed to any group pressure nor tested with conspecifics present (necessary conditions to tap into any form of normative conformity). In light of the absence of conformity in our paradigm where we exposed *knowledgeable* individuals to majority demonstrations, it would be interesting to titrate the effects of increasingly large (relative) majority sizes on chimpanzees’ tendencies to persevere in their first-learned behaviour, especially in relation to first-learned strategies with different magnitudes of familiarity and/or preference (see 20).

 However, the chimpanzees in the present study were not invariably conservative. Instead, most chimpanzees (5/6) showed some evidence of at least trying the alternative strategy when it was upgraded to yield a 5-fold reward compared to their first-learned strategy, with at least half of them converging on this more profitable strategy. Although impossible to quantify the force of the number of demonstrators in comparison to the force of the net increase in the number of peanuts, these findings seem to indicate that for chimpanzees maximizing personal gains provides a stronger motivation to adjust behaviour than matching the majority. Contrary to previous studies showing that chimpanzees did not readily switch to more efficient or rewarding strategies (e.g. [24,25]), this upgrading behaviour matches the predictions from optimal foraging theory, where animals are expected to gradually adjust their foraging behaviour based on the net payoffs of their endeavours [2,3]. In line with theoretical predictions about the usage of social learning strategies [5,33], the chimpanzees could also have employed a selective *copy-if-better* strategy [33,34]. Although our study was not designed to distinguish between specific optimization heuristics (see 34), the chimpanzees central to Study 2 could have copied the behavioural choices of the chimpanzees that were trained on the upgraded location (the three minority subjects from Study 1b) and who were thus rewarded substantially more for exchanging a token. In support of this explanation is the observation that some individuals who upgraded during Study 2 had never tried that vending machine when the payoffs were equal. This might indicate that social demonstrations of the greater efficiency of the alternative strategy (e.g. in form of prolonged presence, food grunting or indirect cues like increased amounts of peanut shells) in the second study were necessary for the chimpanzees to switch strategies. The underlying mechanism could have been relatively simple, where local enhancement and response facilitation would have directed the chimpanzees towards the more efficient strategy. Alternatively, the chimpanzees could have discovered the better strategy by individual exploration. In a follow-up study it would be informative to include a condition in which there is no social reference to the better strategy in order to draw conclusions on the mechanism underlying the behavioural upgrading. 

 One of the switching chimpanzees, however, radically reverted back to his first-learned strategy (i.e. the least productive one) during the last two days of Study 2 (Individual 5; see Figure S2). Albeit counterintuitive, we found indications that this strategy was a payoff-maximizing strategy for this low-ranking individual. Specifically, this individual was the youngest and lowest ranking individual that switched to the most profitable strategy, which caused him to be at risk of losing his peanuts to the more dominant individuals. Together with one young, low-ranking individual that was trained to use the strategy that was upgraded in Study 2, this switching individual was the only one who started to get harassed by others as of day 7 of Study 2 (which was reflected in quick approaches when the low-ranking individuals were getting close to the profitable vending machine, not in theft of the distributed tokens, see Video S1). Since the more profitable vending machine rewarded five peanuts instead of one, there was more time for the dominant individuals to approach these low-ranking individuals after they had inserted a ball in the vending machine and still be successful at stealing one or more rewards. Over time, this pattern of behaviour appeared to cause both these low-ranking individuals to solely use the least profitable vending machine, which in case of individual 5 meant a reversion back to using his first-learned strategy.

 In sum, the knowledgeable state of the individuals in our first study (i.e. being trained on one of the two equally effortful strategies) allowed us to pit chimpanzees’ conservative disposition [24,25] against their postulated tendency to adopt majority strategies [16,17]. Using two complementary designs in which we disentangled the tendency to persevere in using first-learned strategies from conformity and verified that minority chimpanzees actually perceived conflicting social information from the majority of group members (see 20), this study indicates that chimpanzees may not readily conform to majorities, contrary to previous claims [16-18]. In contrast, in the second study, the increased efficiency of the alternative strategy did induce behavioural adjustment in the chimpanzees, which seems to indicate that chimpanzees are more inclined to abandon familiar behaviour for reward maximizing heuristics than for majority biased heuristics. Moreover, the observation that chimpanzees discard ingrained behavioural patterns for better alternatives (this study) calls into question the argument that chimpanzees lack cumulative culture owing to their conservative nature (see 35). This questioning is supported by recent evidence showing that chimpanzees continued exploring a puzzle-box after mastering a reliably rewarding strategy [29], and upgraded their first-learned straw-handling technique (“straw-dipping”) after observing a conspecific using the more efficient “straw-sucking” technique in a juice-foraging task [30].

 Importantly, we note that our studies are inevitably limited in their generalizability. For instance, the selection pressures in a wild setting may place much higher incentives on conforming to the majority of the group than in a setting where the chimpanzees are being provisioned. In a similar vein, the reason why the chimpanzees in our study did upgrade to more profitable strategies (while they have been shown to be reluctant to upgrade in other studies) might be explained by specific characteristics of our study designs. For instance, in our study, the chimpanzees could easily perceive the more profitable strategy because the strategies were spatially separated (cf. [16]). Additionally, they were tested within their social group, which may provide a more relaxed learning environment for chimpanzees than in observation rooms separated from their group (cf. [25]). Also, the chimpanzees in our studies used behaviours that were similar across subgroups: only the token-type (Study 1a) and the location of exchange (Study 1b) differed between individuals, not the actual technique of performance. It could be the case that chimpanzees respond differently to majority influences and superior reward contingencies when the conflicting strategies comprise structurally different techniques, as seems to be indicated by their tool use- and social custom convergence ([13,14], respectively), and the relative absence of upgrading behaviour when the more profitable strategy comprises learning a new technique [24,25]. Similarly, it would be interesting to investigate to what extent the process by which the first strategy was acquired affects chimpanzees’ tendency to adopt better alternatives: Where the chimpanzees in our study had learned their first strategy by means of individual learning, the chimpanzees in the conformity study by Hopper and colleagues had learned their first strategy socially [16]. It might be possible that chimpanzees are more flexible with individually-acquired information than with information that was obtained by observing conspecifics, which seemed to be demonstrated in a study by Price and colleagues, where chimpanzees who had learned to use a raking tool socially were adjusting the tool less efficiently than the chimpanzees who had discovered the use of the tool by themselves [36].

 In this study, we found chimpanzees to be more motivated to maintain their first-learned strategy than to conform to the majority of group members. The presence of a superior alternative, however, did sever their fidelity to their first-learned strategy, indicating that chimpanzees selectively adjust their behaviour, given the right kind of incentive. These findings demonstrate that chimpanzees, albeit sensitive to social influences in many contexts, weigh their own knowledge and experience heavily in the process of decision making. Exploring the points of bifurcation in animals’ learning dynamics is an exciting endeavour and continues to be a fruitful enterprise for gaining insights in species-specific behaviour, the extent to which learning biases are distributed across taxa, and which selection pressures might have given rise to their existence.

## Supporting Information

Figure S1 **Depicted are the vending machines used in Study 1b and Study 2: The Plexiglas structure with the automated peanut dispenser (a), and the painted metal frames with the corresponding holes for the wooden balls (hole Y) and the food rewards (hole X)**. The “Princes Bakery” (b) and the “G&G Bakery” (c) were the trained strategies for the majority and minority, respectively, where the latter was upgraded in Study 2.
(TIF)

Figure S2 **Individual preferences for the trained strategy of the majority chimpanzees in Zambia throughout Study 2.** **Data points at time point “1-10” represent the average preferences for the trained strategy over the first 10 days per individual (Study 1b)**. Data points at time points 11 to 20 refer to the individual preferences for the trained strategy (the least profitable) compared to the non-trained strategy (the most profitable).
(TIF)

Table S1 **Subgroups (majority and minority) in Study 1a (Leipzig) and 1b (Zambia).** **Individuals who actually participated during the test-sessions are designated in bold; kinship relations are indicated by matching symbols. Rank was categorized by the alpha male (“1”) and three categories (High, Middle, and Low) based on keeper reports and personal observations**. The majority individuals in Zambia were the focus individuals for Study 2, where the minority strategy was upgraded in terms of rewards.
(DOCX)

Table S2 **Minority (**m**) and Majority (**M**) responses of the minority chimpanzees in the Leipzig and Zambia groups across the 10 days of Study 1 (conformity study: equal rewards for both strategies).** Minority responses equal the responses that the minority chimpanzees were trained on (Leipzig: white token; Zambia: G&G Bakery).
(DOCX)

Video S1 **An adolescent male chimpanzee named Kit (7 years old), heads diagonally for the most profitable vending machine (G&G Bakery; 5 peanuts/token) with a token (wooden ball) in his mouth when he seemingly gets interrupted by the movements of two adult males and diverts towards the least profitable vending machine (Princes Bakery; 1 peanut/token), where he subsequently exchanges his token.** This video was taken at the Chimfunshi Wildlife Orphanage Trust on the 9^th^ day of Study 2.
(MPG)
