# Supplementary figures and images for: Chimpanzees (Pan troglodytes) Flexibly Adjust Their Behaviour in Order to Maximize Payoffs, Not to Conform to Majorities
Source: PLoS One. 2013 Nov 27;8(11):e80945. doi: 10.1371/journal.pone.0080945 (PMC3842352; doi:10.1371/journal.pone.0080945)

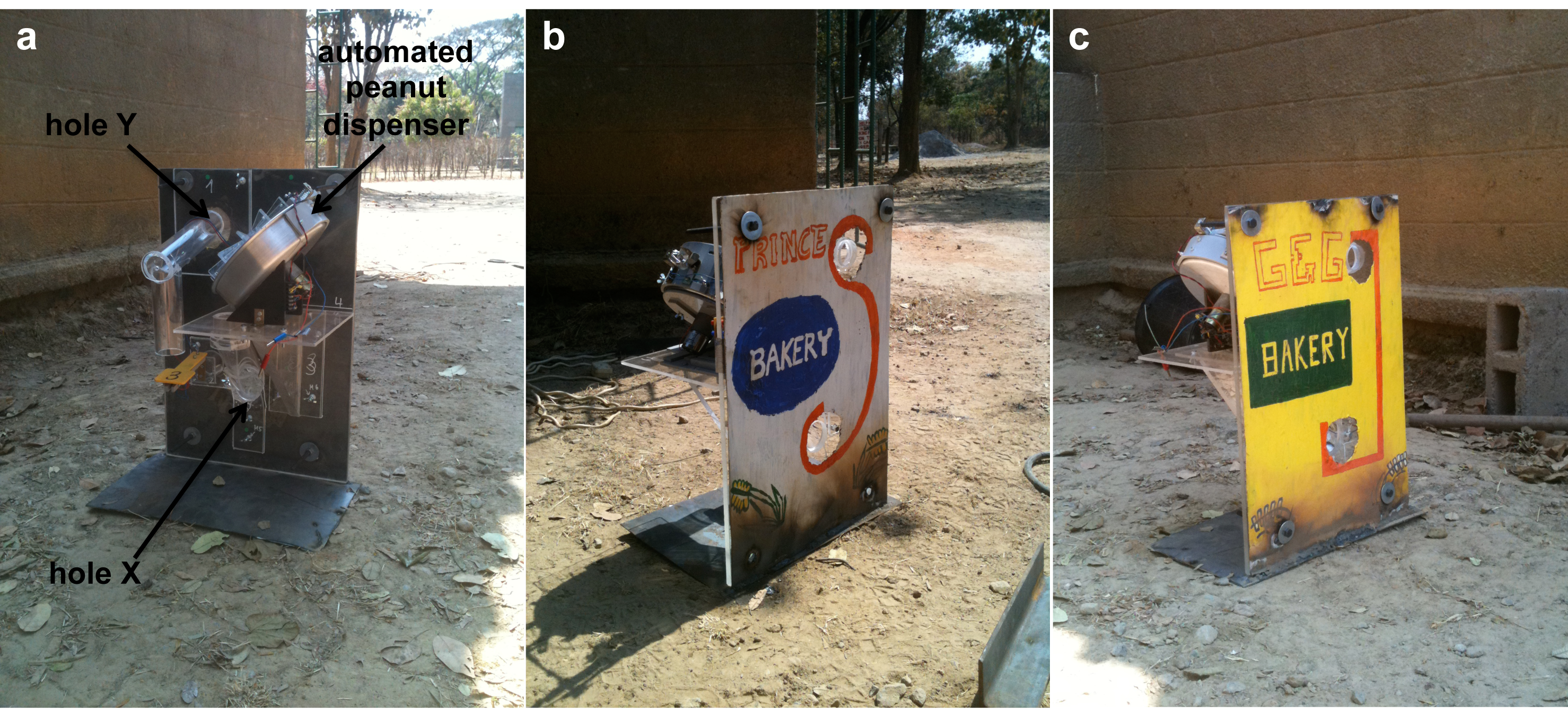

Supplement: Figure S1 — Depicted are the vending machines used in Study 1b and Study 2: The Plexiglas structure with the automated peanut dispenser (a), and the painted metal frames with the corresponding holes for the wooden balls (hole Y) and the food rewards (hole X). The “Princes Bakery” (b) and the “G&G Bakery” (c) were the trained strategies for the majority and minority, respectively, where the latter was upgraded in Study 2. (TIF) [file pone.0080945.s001.tif]

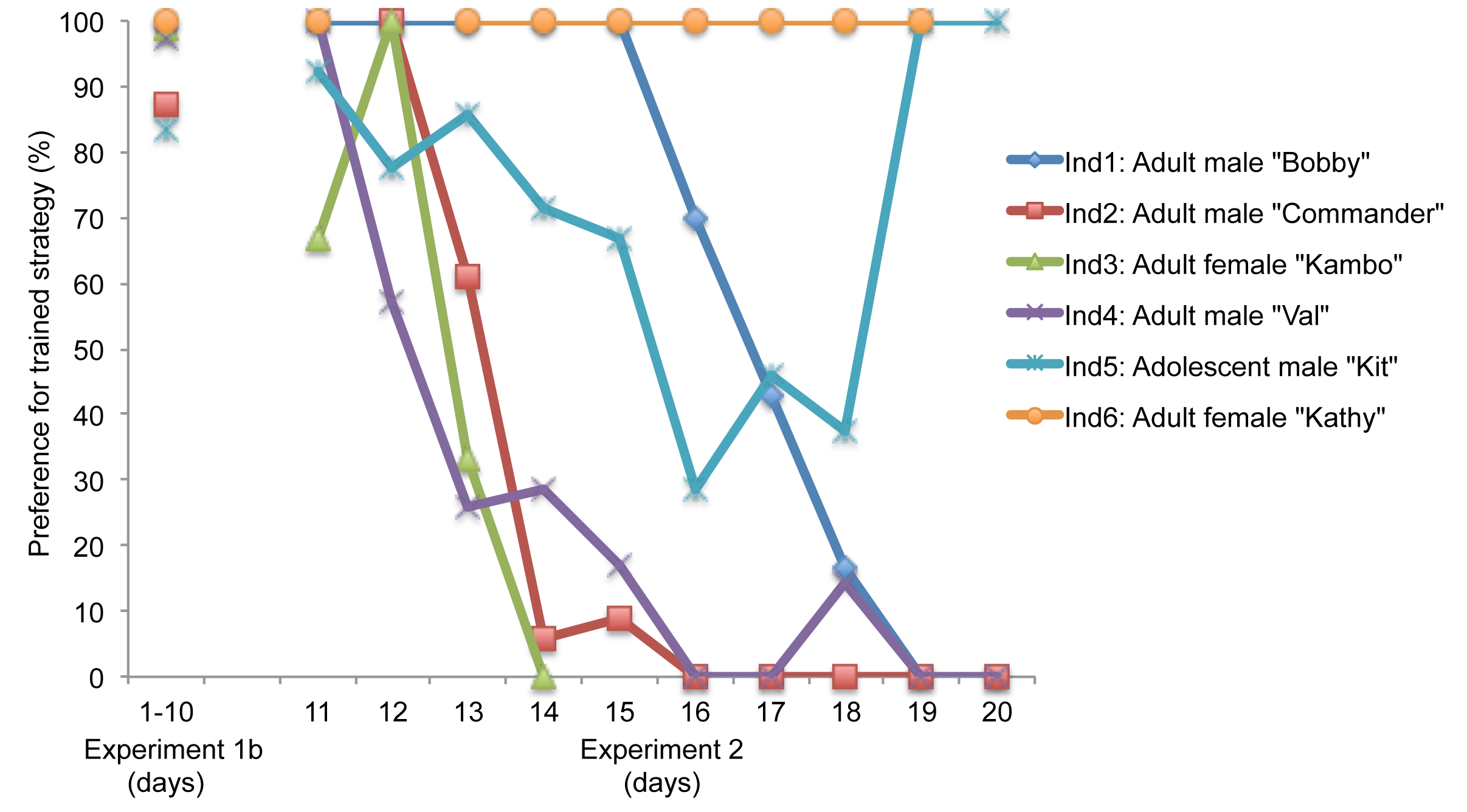

Supplement: Figure S2 — Individual preferences for the trained strategy of the majority chimpanzees in Zambia throughout Study 2. Data points at time point “1-10” represent the average preferences for the trained strategy over the first 10 days per individual (Study 1b). Data points at time points 11 to 20 refer to the individual preferences for the trained strategy (the least profitable) compared to the non-trained strategy (the most profitable). (TIF) [file pone.0080945.s002.tif]
