# Supplementary material for: Chimpanzees (Pan troglodytes) Flexibly Adjust Their Behaviour in Order to Maximize Payoffs, Not to Conform to Majorities
Source: PLoS One. 2013 Nov 27;8(11):e80945. doi: 10.1371/journal.pone.0080945 (PMC3842352; doi:10.1371/journal.pone.0080945)
Supplement: Table S1 — Subgroups (majority and minority) in Study 1a (Leipzig) and 1b (Zambia). Individuals who actually participated during the test-sessions are designated in bold; kinship relations are indicated by matching symbols. Rank was categorized by the alpha male (“1”) and three categories (High, Middle, and Low) based on keeper reports and personal observations. The majority individuals in Zambia were the focus individuals for Study 2, where the minority strategy was upgraded in terms of rewards. (DOCX) [file pone.0080945.s003.docx]

**Table S1**. A priori distribution of individuals over the subgroups (majority and minority) in Experiment 1a (Leipzig) and 1b (Zambia). Individuals who actually participated during the test-sessions are designated in bold; kinship relations are indicated by matching symbols. Rank was categorized by the alpha male (“1”) and three categories (High, Middle, and Low) on the basis of keeper reports and personal observations. The majority individuals in Zambia were the focus individuals for Experiment 2, where the minority strategy was upgraded in terms of rewards.

| **Group** | **Majority** | **Rank** | **Sex** | **Age** | **Minority** | **Rank** | **Sex** | **Age** |
| --- | --- | --- | --- | --- | --- | --- | --- | --- |
| Leipzig | Robert | H | male | 36 | Corry^★^ | H | female | 35 |
|  | **Fraukje**^◼^ | L | female | 35 | Sandra | M | male | 18 |
|  | **Ulla^✜^** | H | female | 34 | Frodo | 1 | female | 18 |
|  | Riet^❖^ | H | female | 34 | **Tai**^❖^ | M | female | 9 |
|  | Natascha | M | female | 31 | **Lobo**^★^ | L | male | 7 |
|  | **Dorien** | H | female | 31 |  |  |  |  |
|  | **Swela** | M | female | 16 |  |  |  |  |
|  | **Pia** | L | female | 12 |  |  |  |  |
|  | **Lome**^★^ | M | male | 10 |  |  |  |  |
|  | **Kara**^◼^ | L | female | 6 |  |  |  |  |
|  | **Kofi^✜^** | L | male | 6 |  |  |  |  |
| Zambia | **Bobby** | L | male | 19 | **Nicky** | M | male | 21 |
|  | Julie^✪^ | H | female | 18 | Sinky | L | male | 18 |
|  | **Kambo**^◆^ | L | female | 16 | **Miracle** | H | female | 11 |
|  | **Kathy** | H | female | 13 | **Jack**^✪^ | L | male | 5 |
|  | **Commander** | 1 | male | 12 |  |  |  |  |
|  | Berta | L | female | 12 |  |  |  |  |
|  | **Val** | M | male | 11 |  |  |  |  |
|  | **Kit**^◆^ | L | male | 7 |  |  |  |  |
