# Supplementary material for: Chimpanzees (Pan troglodytes) Flexibly Adjust Their Behaviour in Order to Maximize Payoffs, Not to Conform to Majorities
Source: PLoS One. 2013 Nov 27;8(11):e80945. doi: 10.1371/journal.pone.0080945 (PMC3842352; doi:10.1371/journal.pone.0080945)
Supplement: Table S2 — Minority (m) and Majority (M) responses of the minority chimpanzees in the Leipzig and Zambia groups across the 10 days of Study 1 (conformity study: equal rewards for both strategies). Minority responses equal the responses that the minority chimpanzees were trained on (Leipzig: white token; Zambia: G&G Bakery). (DOCX) [file pone.0080945.s004.docx]

**Table S2.** Minority (m) and Majority (M) responses of the minority chimpanzees in the Leipzig and Zambia groups across the 10 days of experiment 1 (conformity study: equal rewards for both strategies). Minority responses equal the responses that the minority chimpanzees were trained on (Leipzig: white token; Zambia: G&G Bakery).

|  |  |  | **Day 1** | | **Day 2** | | **Day 3** | | **Day 4** | | **Day 5** | | **Day 6** | | **Day 7** | | **Day 8** | | **Day 9** | | **Day 10** | |
| --- | --- | --- | --- | --- | --- | --- | --- | --- | --- | --- | --- | --- | --- | --- | --- | --- | --- | --- | --- | --- | --- | --- |
| **Group** | **Subject** | **Sex** | m | M | m | M | m | M | m | M | m | M | m | M | m | M | m | M | m | M | m | M |
| Leipzig | Tai | Female | 30 | 10 | 28 | 6 | 81 | 2 | 54 | 2 | 50 | 7 | 58 | 9 | 43 | 2 | 56 | 5 | 39 | 8 | 55 | 2 |
|  | Lobo | Male | 2 | 0 | 4 | 0 | 11 | 0 | 18 | 0 | 40 | 0 | 32 | 0 | 27 | 0 | 37 | 0 | 33 | 0 | 23 | 0 |
| Zambia | Nicky | Male | 3 | 0 | 9 | 0 | 10 | 0 | 5 | 0 | 4 | 0 | 3 | 0 | 0 | 0 | 35 | 0 | 39 | 0 | 10 | 0 |
|  | Miracle | Female | 0 | 0 | 0 | 0 | 1 | 0 | 2 | 0 | 6 | 0 | 13 | 1 | 0 | 0 | 8 | 2 | 5 | 2 | 9 | 0 |
|  | Jack | Male | 6 | 0 | 8 | 3 | 0 | 0 | 4 | 1 | 11 | 2 | 15 | 3 | 22 | 3 | 6 | 1 | 3 | 3 | 4 | 0 |
